# Effect of Adding Extra Virgin Olive Oil to Hair Sheep Lambs’ Diets on Productive Performance, Ruminal Fermentation Kinetics and Rumen Ciliate Protozoa

**DOI:** 10.3390/ani12192588

**Published:** 2022-09-28

**Authors:** Darwin N. Arcos-Álvarez, Edgar Aguilar-Urquizo, José R. Sanginés-García, Alfonso J. Chay-Canul, Isabel Molina-Botero, Magnolia Tzec-Gamboa, Einar Vargas-Bello-Pérez, Ángel T. Piñeiro-Vázquez

**Affiliations:** 1 Tecnológico Nacional de México, Campus Conkal, Avenida Tecnológico s/n, Conkal 97345, Yucatán, México; 2 División Académica de Ciencias Agropecuarias, Universidad Juárez Autónoma de Tabasco, Carretera Villahermosa-Teapa, km 25, Villahermosa 86280, Tabasco, México; 3 Departamento de Nutrición Animal, Facultad de Zootecnia, Universidad Agraria La Molina, La Molina, Lima 15024, Perú; 4 Facultad de Medicina Veterinaria y Zootecnia, Universidad Autónoma de Yucatán, C.P., Mérida 97300, Yucatán, México; 5 Department of Animal Sciences, School of Agriculture, Policy and Development, University of Reading, P.O. Box 237, Earley Gate, Reading RG6 6EU, UK

**Keywords:** energy, vegetable oils, supplementation, conjugated linoleic acid, ruminants

## Abstract

**Simple Summary:**

The use of added lipids in the diets of ruminants has been found to have beneficial effects. In this study, the effects of different doses of extra virgin olive oil on the productive traits and ruminal fermentation parameters in lambs were evaluated. The relationship between nutrient intake and digestibility was optimal with 2% oil inclusion. The concentration of propionic acid increased with 2 and 4% DM of olive oil, while butyric acid decreased. The intake of olive oil did not affect the population of protozoa or animal performance. The inclusion of olive oil in low concentrations (2% of DM) positively influences feed intake and nutrient digestibility in hair sheep lambs.

**Abstract:**

This study determined productive performance, ruminal fermentation kinetics and rumen ciliate protozoa in hair sheep lambs fed different levels of olive oil. Twenty-four growing lambs were used, with an initial live weight of 10.5 ± 2.9 kg, and randomly assigned into four treatments (six animals per treatment) containing increasing levels of extra virgin olive oil (0, 2, 4 and 6% of dry matter). Animals were fed for 80 days, and sampling was carried out weekly. Intake of dry matter (DM), organic matter (OM), crude protein (CP), neutral detergent fiber (NDF), acid detergent fiber (ADF) and metabolizable energy (ME) differed between treatments (*p* < 0.05), with a linear and cubic tendency to decrease when oil concentrations were increased. Digestibility coefficients of OM, CP and NDF were not affected; however, the relationship between total intake and nutrient digestibility (DM, OM, NDF, ADF) increased with 2% DM olive oil. Compared with all treatments, the concentration of propionic acid increased by 16% with 4% olive oil. The intake of olive oil did not affect the protozoa population and live weight gain. Overall, the inclusion of olive oil in low concentrations (2% of DM) positively influences feed intake and nutrient digestibility in hair sheep lambs.

## 1. Introduction

In the ruminant diet, cereals and oilseeds are the main sources of lipids such as triglycerides, while glycolipids and phospholipids can be found in forages such as grasses and legumes [1]. However, in tropical and subtropical regions, where sheep diets are mainly composed of grasses of medium to low quality, their energy intake is low (<14 MJ metabolizable energy/kg of dry matter; DM), and this negatively affects the metabolic and productive performance [2]. In this sense, the addition of dietary vegetable oils is used to increase caloric density, reduce the amount of dust, and improve acceptability [3,4,5], as well as improving feed conversion, reducing the feeding costs and increasing production profitability. Additionally, it has been reported that the intake of lipids can increase the productive response and positively change the fatty acid profiles of meat and milk [6,7,8], in addition to reducing enteric methane production [9], and these factors can be considered as added values to the final products.

The above benefits are due to a change in ruminal fermentation patterns [10]. However, sources of vegetable oils such as flaxseed, palm, coconut, soybean and sunflower, among others, must be supplied as protected fats by physical or chemical procedures (encapsulation, biohydrogenation, calcium salts and acylamides), since high doses of these oils in the diet generate a decrease in fibrotic activity [11], especially in protozoa [5]. The composition of olive oil makes it unique within the group of vegetable oils, since it is rich in palmitic (C16:0), palmitoleic (C16:1), stearic (C18:0), oleic (C18:1), linoleic (C18:2) and linolenic (C18:3) acids [12].

In tropical regions, sheep feeding is based on the use of local or introduced forage resources that at certain times of the year have low quality (>7% CP, 10 MJ/kg DM and NDF > 70%) and low availability. In this sense, small and medium-sized producers rely on the use of local resources from the agricultural and livestock industry to increase dietary energy and protein contents. These systems use poultry manure as a source of non-protein nitrogen while frying oil or frying waste are used as energy sources for improving animal performance. However, adding these feedstuffs can have negative consequences on animals’ (i.e., parasite and copper poisoning) and consumers’ health. Therefore, it is important to search for alternative energy sources such as the use of vegetable oils, oilseeds and their by-products.

Besides the information presented above, information on the use of olive oil under tropical conditions and especially as a feeding strategy for lambs is limited. We hypothesized that the inclusion of olive oil in the diet of hair sheep lambs would increase the concentration of propionic acid and consequently improve the productive performance. For this reason, the objective of this study was to determine the effect of different levels of inclusion of olive oil on the productive performance, ruminal fermentation kinetics and rumen ciliate protozoa in hair sheep lambs.

## 2. Materials and Methods

### 2.1. Animals

Lambs were treated in accordance with guidelines and regulations for animal experimentation of the Instituto Tecnológico de Conkal (Project ID ITC: MX-ITC-002521 and NOM-033-SAG/ZOO-2014).

### 2.2. Study Site

The experiment was carried out in the Agricultural and Livestock Production and Research Unit of the Technological Institute of Conkal, located at 21°05′ North latitude and 89°32′ West longitude, at 8 m above sea level, rainfall 900 mm and average annual temperature of 26.5 °C [13].

### 2.3. Experimental Design

Twenty-four crossbred growing male lambs of two months of age were used, with an initial live weight (LW) of 10.5 ± 2.9 kg (mean ± SD), and randomly assigned into four treatments (6 animals per treatment). The lambs were housed in individual (0.80 m × 2.0 m) roofed pens with a raised floor, where they had free access to water. Pens were provided with ceiling fan ventilation. Prior to the start of the trial, they were weighed and dewormed with vermectin (Silvermec^®^ ADE), at a dose of 0.1 mL/5 kg LW, and a vitamin supplement ADE (Vigantol^®^ ADE) was applied intramuscularly. During the study, average temperature was 26 °C and relative humidity was >85%.

The lambs were weighed at the beginning of the study and subsequently every 8 days for 80 days, to adjust their diet and obtain a rejection greater than 10% to consider ad libitum feeding. Weighing was performed using an electronic platform scale with a capacity of 300 kg with a margin of error of 0.100 kg (OHAUS^®^-T31P, Mexico).

### 2.4. Dietary Treatments

Four treatments with increasing levels of olive oil were added to a basal diet: 0, 2, 4 and 6% dry matter (DM). During feed preparation, specifically in the mixing process, extra virgin olive oil was added to the diet as part of the energy ingredients. The olive oil was extra virgin, and its fatty acid profile is shown in Table 1.

The diets were prepared according to the requirements established by the AFRC [14], for supplying estimated metabolizable energy of 12 MJ and 16% of crude protein per kg of DM estimated for a weight gain of 220 g/day (Table 2). The daily ration (40:60 forage: concentrate) corresponded to 5.5% of body weight and was composed of corn stover (40%), ground corn (18.08%), soybean hay (19.82%), wheat bran (10%), molasses (10%), mineral mixture (2%) and Vitamin ADE (0.1%), respectively. Prior to the study, the animals were adapted for a period of 15 days to handling and diet.

### 2.5. Sampling

#### 2.5.1. Productive Traits

Total weight gain (TWG) was calculated as the difference between the final weight (FW) minus the weight at the start (IW) of the experiment = (FW-IW). Daily weight gain (DWG) was determined by dividing TWG by days in the trial = (TWG)/(D). Feed conversion (FC) was determined by dividing feed intake by daily weight gain = (VI)/(DWG).

#### 2.5.2. Feed Intake

Animals were fed ad libitum, allowing a rejection of less than 10% of the dry matter offered the previous day. Feed intake was determined by the difference between the amount offered and rejected. Feed rejection was weighed at 0900 h the next day.

#### 2.5.3. Determination of Rumen Fluid pH and Volatile Fatty Acids

Rumen fluid samples were taken through an esophageal tube according to the technique proposed by Ramos-Morales et al. [15]. Rumen fluid was obtained once a week, six postprandial hours according to the recommendations of Hales et al. [16] and Bhatta et al. [17], to measure rumen fluid pH and analyze volatile fatty acids (VFA). The ruminal fluid samples were filtered through a double layer of gauze to retain large particles, and the pH was immediately measured with a portable potentiometer (HANNA Instruments, Woonsocket, RI, USA), previously calibrated with buffer solutions with a pH of 4, 7 and 10. Then, 4 mL of metaphosphoric acid was added to one milliliter of ruminal liquid to later determine the production of VFA. The VFA determination was carried out using the technique proposed by Ryan [18] using a gas chromatograph (Hewlett-Packard, 5890 series III, CA, USA), equipped with a flame ionization detector (FID). The type of column used was HP-FFAP of 30 m × 0.53 mm, the injector temperature was 200 °C and the detector temperature was 200 °C.

#### 2.5.4. Protozoan Population

The protozoan count was carried out according to the procedure described by Rosales [19], making a 1:1 mixture of ruminal fluid (sample extraction once a week) and saline solution of methyl green formalin (35 mL/L of formaldehyde, 0.14 mM NaCl, 0.92 mM methylgreen) and then centrifuged at 2000 rpm for 20 min. Then, an aliquot was taken to be introduced into a Neubauer improved chamber (Tiefe depth 0.100 mm) and observed under a microscope (Leica-DM500) at 40×. The number of protozoa was estimated as follows: number of cells per mL^−1^ = [(n1 + n2 + n3 + n4 + n5)/5]/0.022 mm^3^ × 10^3^ × d, where n1 … n5: number of protozoa per large square and d = dilution factor [20]. The classification of the protozoa was carried out according to Ogimoto and Imai [21].

#### 2.5.5. Apparent Digestibility

The apparent digestibility of dry matter, organic matter, crude protein, neutral detergent fiber and acid detergent fiber was performed using the total feces collection method [22], taking a subsample of feces (10%) per day. Subsequently, the sample was kept refrigerated until chemical analysis.

### 2.6. Chemical Analysis

Determination of DM of feed was carried out in a forced-air oven at 55 °C for 48 h (constant weight) (#7.007) [23]. The protein content was found from the N content (CP = N × 6.25) that was carried out by combustion using LECO CN2000 series 3740 equipment (LECO, Corporation, #2.057) [23]. Likewise, the content of organized matter was obtained from the ash content determined by incineration in a muffle at 550 °C for 6 h (AOAC Method #923.03) (OM = 100 - Ash, %) and the fiber content in neutral and acid detergent fiber was determined as suggested by Van Soest et al. [24]. The fatty acid profile of olive oil was determined by gas chromatography as proposed by Ryan et al., [18], in which a gas chromatograph (Hewlett-Packard, 5890 series III) equipped with a gas chromatograph, flame ionization detector (FID) and a 30 m × 0.53 mm HP-FFAP column were used. The temperature of the injector and the detector was 200 °C.

### 2.7. Statistical Analysis

The data were analyzed using a completely randomized design by analysis of variance considering the treatments as fixed effects and the lamb as the random effect [25]. Tukey’s test was performed when a significant treatment effect was detected. Additionally, a surface response analysis was carried out to assess the linear, quadratic or cubic effects of the response to treatments (0, 2, 4 and 6% of olive oil in the ration) [25]. Significant differences were declared at *p* < 0.05.

## 3. Results

### 3.1. Intake and Apparent Digestibility

Dry matter (DM) intake differed between treatments (*p* < 0.05) and had a linear tendency to decrease when olive oil was added at higher concentrations (Table 3). This pattern was similar for CP, NDF and ADF intake (*p* < 0.05). Contrary to what occurred with 4% oil, the apparent digestibility of ADF was favored by the addition of 2% oil (Table 3). The digestibility coefficients of OM, CP and NDF were not influenced by the addition of olive oil (*p* > 0.05). There were differences between the levels of oil supplied for the intake of nutrients, with a quadratic trend for digestible CP, while the digestible intake of OM and NDF decreased linearly when higher levels of oil were included. The intake of metabolizable energy was higher (*p* < 0.05) with 2% oil, and except for CP, this was similar to the intake of digestible nutrients.

### 3.2. PH and Molar Ratios of Volatile Fatty Acids in the Rumen

Rumen pH was not affected by the addition of olive oil (Table 4), and the same result was observed in the molar proportion of acetic acid (*p* > 0.05). Adding 4% oil resulted in the highest concentration of propionic acid. For butyric acid, an inverse quadratic pattern to propionic acid was observed (*p* < 0.05) since the highest production of this fatty acid occurred with the minimum and maximum level of dietary oil inclusion (0 and 6%).

### 3.3. Rumen Protozoa

Populations of Holotrichs and Entodinium and the total number of protozoa were not affected by the different levels of addition of olive oil in the diet (*p* > 0.05), as observed in Table 5.

### 3.4. Productive Performance

Daily weight gain was not affected by treatments but olive oil at 0, 2, 4 and 6% increased (*p* > 0.05) daily weights (0.17, 0.18, 0.18 and 0.16 kg/day, respectively). There were no linear, quadratic or cubic effects (Table 6).

## 4. Discussion

### 4.1. Intake and Apparent Digestibility of Nutrients

Feed intake is one of the most important indicators of animal behavior, and various factors can affect feed intake, such as the level of protein and fiber or the addition of ingredients such as oils. The variation in nutrient intake and digestibility may be related to the type or amount of oil supplied [26]. This was corroborated in the present study, since the highest DM intake was obtained with the addition of only 2% olive oil, which is similar to that obtained by Najafi et al. [27], who reported positive effects between the intake and the addition of 2% palm oil and soybean oil, observing intakes of 1.15 and 1.06 kg of DM per day, respectively, and those values were similar to those found in this study (1.02 kg DM/day on average). Similarly, Van-Cleef et al. [28] found negative effects on DM intake with the addition of 6% soybean oil in the diet of crossbred lambs, and Roy et al. [29] reported a reduction in intake when soybean and sunflower oil were supplied at 4.5% in goat diets.

There are studies where an inverse relationship between oil intake and fiber degradation has been reported; for example, Van-Cleef et al. [28] found a reduction in the digestibility of DM and fiber with an inclusion level of 6% of vegetable oils. These results are similar to those found in the present study, since a reduction in digestible intake of NDF was observed in the two maximum inclusion levels of olive oil (4 and 6%). This can be explained as indicated by Cobellis et al. [30], by a reduction in the population of protozoa and cellulolytic bacteria such as Fibrobacter succinogenes, Ruminococcus albus and Ruminococcus flavefaciens. Similarly, the reduction in the intake of degraded crude protein due to the inclusion of olive oil may be due to a decrease in the group of bacteria responsible for carrying out the breakdown of this compound at the rumen level [30].

### 4.2. Molar Proportions of Volatile Fatty Acids and pH in the Rumen

The molar proportion of volatile fatty acids is modified according to the type of substrate that reaches the rumen and indicates the efficiency in the fermentation of nutrients. The effect of some vegetable oils on VFA production is variable. For example, an increase has been reported in the concentration of acetic acid [31] or in the production of propionic acid [32], or no changes were reported [33]. These results are associated with the time of supplementation of vegetable oils in the diet [34] and the offered dose of oils. For example, in this study, it was found that at low and intermediate doses, the proportion of propionic acid increases, while above 4% inclusion, the production of propionic acid is depressed; quite the opposite was observed with butyric acid.

In this study, changes in VFA production are directly related to the effect of oils on ruminal pH [35]. According to Spanghero et al. [36], the production of propionic acid is favored by slightly low pH. However, in our study, despite showing changes in the concentration of propionic acid in the rumen, the ruminal pH was not affected by the inclusion of olive oil; on the contrary, it was within the optimal values (7 ± 0.5) for ruminal microorganisms [37,38]. It is possible that the amount of fiber included in the diet of the lambs (40%) maintained a pH close to 7, which indicates a diet with a high proportion of cellulose with a higher population of cellulolytic bacteria responsible for degrading the fibrous fractions of the food and which generate an acetic fermentation pattern.

### 4.3. Population of Protozoa in the Rumen

The changes that are generated in the ruminal environment depend on diet composition and the amount and the type of dietary lipids [39], which is why the effect of the oils on the number and/or activity of the protozoa is variable [40]. In previous studies, Ivan et al. [41], Cieslak et al. [42] and Majewska et al. [43] reported a decrease in the total population or in some species of protozoa such as Entodinium, by supplying between 5.2 and 6% of sunflower, rapeseed, flaxseed or fish oils in cows and sheep diets. In the present study, the amount of the protozoan population was not affected by the addition of dietary olive oil; however, it is likely that the activity of this species decreased, and this is explained in part by the reduction in the fiber degradation. According to Matsumoto et al. [44], lauric (C12) and myristic (C14) acids, which are found in olive oil, have negative effects on protozoa membranes, and this can lead to a reduction in their fibrolytic activity. The inclusion of dietary unprotected olive oil in lambs should be further studied, analyzing rumen microbiome and obtaining more comprehensive details on how olive oil affects different microorganism populations.

### 4.4. Productive Performance

Our results are similar to those reported by Liu et al. [32], who incorporated 2.5% coconut oil without affecting ADG, feed intake and CA in sheep, and González et al. [45], who carried out trials with different oils (flaxseed, sunflower and soybean) in Rubia Gallega calves, adding 4.5% oils. The above results contradict those obtained by Van-Cleef et al. [28], who added 6% soybean oil to the diet of sheep crossbreeds or De la Fuente et al. [46] with the addition of 3.3% palm oil in the feeding of lambs of the Manchego breed, and in the same way, Bhatt et al. [47] reported a positive effect on daily gain by adding 2.5, 5.0 and 7.5% coconut oil to the diet. In this study, it was observed that 4% olive oil inclusion leads to increases in propionic acid. However, this had no effect on daily weight gain, which could be related to the lower intake and digestibility of some nutrients, and these two factors are important for increasing or reducing weight gains and feed conversion. It is possible that the reduction in feed intake is related to feed acceptability; however, several studies mentioned that the limit of dietary inclusion of olive oil is 6% DM [28], as this level negatively affects ruminal protozoa and cellulolytic bacteria responsible for fermentation and ruminal degradation of nutrients [30]. All these factors can lead to a reduction in nutrient digestibility with negative consequences on productive performance.

## 5. Conclusions

The dietary inclusion of olive oil at 4 and 6% DM reduced nutrient intake in growing lambs and affected apparent digestibility coefficients of ADF. The relationship between nutrient intake and digestibility (DM, OM, NDF, ADF) was optimal with 2% oil inclusion. The concentration of propionic acid increased with 2 and 4% DM olive oil, while butyric acid decreased. The intake of olive oil did not affect the protozoa population or animal performance. Based on these results, it is suggested to continue this research with measurements of enteric methane, carcass characteristics and the transfer of fatty acids such as oleic acid into meat fat from lambs.

## Figures and Tables

**Table 1:** Fatty acid profile of extra virgin olive oil used to feed growing lambs.

Fatty Acid	g/100 g of Detected Fatty Acids
C6:0	0.34
C8:0	0.33
C11:0	0.17
C12:0	0.36
C13:0	0.24
C14:0	0.45
C14:1	0.28
C15:0	0.23
C15:1	0.23
C16:0	0.67
C16:1	0.79
C17:0	0.21
C17:1	0.34
C18:0	0.44
C18:1 cis9	69.5
C18:2n6t	19.1
C18:2n6c	0.21
C20:0	2.15
C18:3n6	0.27
C20:1	0.29
C18:3n3	0.22
C22:0	0.30
C20:3n6	0.23
C22:1n9	0.23
C20:3n3	0.39
C20:4n6	0.16
C23:0	0.21
C22:2	0.22
C24:0	0.44
C20:5n3	0.78
C24:1	0.10

**Table 2:** Ingredients and chemical composition of dietary treatments fed to growing lambs.

Ingredients, %	Olive Oil, % DM			
	0	2	4	6
Corn stover	40	40	40	40
Ground corn	18.0	18.0	18.0	18.0
Soybean hay	19.8	19.8	19.8	19.8
Wheat bran	10.0	10.0	10.0	10.0
Molasses	10.0	10.0	10.0	10.0
Mineral mixture	2.0	2.0	2.0	2.0
Vitamin ADE	0.1	0.1	0.1	0.1
Olive oil	-	2.0	4.0	6.0
Chemical analysis, % dry matter				
Dry matter	90.6	90.6	90.6	90.6
Organic matter	7.7	7.62	7.62	7.62
Ashes	92.3	92.3	92.3	92.3
Crude protein	16.1	15.8	16.5	16.3
Acid detergent fiber	24.5	23.3	24.5	25.1
Neutral detergent fiber	43.1	43.1	43.1	43.1
Ether extract	2.05	4.05	6.05	8.05
Total fatty acids (g/100 g)	0.65	11.799	22.61	33.1
Nitrogen-free extract (%)	21.65	20.03	17.33	15.53

**Table 3:** Intake and apparent digestibility of lambs fed with different levels of olive oil.

Items	Olive Oil % DM					Significance			
							Contrast		
	0	2	4	6	SE	*p*-Value	L	Q	C
Live weight (kg)	18.9	19.2	19.3	17.3	0.65	0.107	0.102	0.080	0.530
Metabolic live weight (kg)	9.02	9.10	9.17	8.45	0.23	0.121	0.120	0.090	0.457
Intake (kg/d)									
DM	0.96 ^ab^	1.02 ^a^	0.82 ^c^	0.86 ^bc^	0.02	<0.001	0.002	0.800	<0.001
DM (% LW)	5.40 ^ab^	6.06 ^a^	4.34 ^c^	5.19 ^b^	0.21	<0.001	0.016	0.662	<0.001
DM (g/kg ^0.75^)	110 ^ab^	121 ^a^	90.2 ^c^	104 ^b^	3.79	<0.001	0.004	0.728	<0.001
OM	0.73 ^ab^	0.78 ^a^	0.63 ^c^	0.66 ^bc^	0.02	<0.001	<0.001	0.801	<0.001
CP	0.15 ^ab^	0.16 ^a^	0.13 ^c^	0.14 ^bc^	0.004	<0.001	<0.001	0.801	<0.001
NDF	0.41 ^ab^	0.44 ^a^	0.35 ^c^	0.37 ^bc^	0.01	<0.001	<0.001	0.801	<0.001
ADF	0.23 ^ab^	0.25 ^a^	0.20 ^c^	0.21 ^bc^	0.007	<0.001	<0.001	0.806	<0.001
ME (MJ/day)	9.98 ^ab^	11.1 ^a^	8.71 ^bc^	7.57 ^c^	0.56	0.032	<0.001	0.027	0.027
Apparent digestibility (kg/d)									
DM	0.64	0.69	0.64	0.68	0.01	0.093	0.514	0.713	0.014
OM	0.60	0.64	0.59	0.59	0.02	0.257	0.473	0.415	0.110
CP	0.74	0.78	0.76	0.77	0.01	0.455	0.309	0.323	0.286
ADF	0.46 ^ab^	0.56 ^a^	0.44 ^b^	0.49 ^ab^	0.02	0.023	0.806	0.428	0.003
NDF	0.57	0.64	0.55	0.60	0.02	0.073	0.997	0.718	0.010
Digestible intake (kg/day)									
DMD	0.83 ^ab^	1.01 ^a^	0.76 ^b^	0.78 ^b^	0.03	<0.001	0.060	0.058	<0.001
OMD	0.63 ^ab^	0.70 ^a^	0.55 ^bc^	0.48 ^c^	0.02	<0.001	<0.001	0.027	0.027
CPD	0.18 ^a^	0.10 ^c^	0.14 ^b^	0.14 ^b^	0.007	<0.001	0.016	<0.001	<0.001
NDFD	0.33 ^ab^	0.39 ^a^	0.30 ^bc^	0.28 ^c^	0.01	<0.001	0.005	0.028	0.004
ADFD	0.14 ^ab^	0.20 ^a^	0.13 ^b^	0.14 ^b^	0.009	<0.001	0.089	0.060	<0.001

^a, b, c^ Columns with different letters indicate statistical difference (*p* < 0.05); SE: standard error; L: linear; Q: quadratic; C: cubic; DM: dry matter; OM: organic matter; CP: crude protein; NFD: neutral detergent fiber; ADF: acid detergent fiber; DMD: digestible dry matter; OMD: digestible organic matter; CPD: digestible crude protein; NDFD: digestible neutral detergent fiber; ADFD: digestible acid detergent fiber. kg^0.75^= metabolic weight.

**Table 4:** Effect of the inclusion of olive oil on rumen fluid pH and molar proportion of volatile fatty acids.

Items	Olive Oil % DM					Significance			
							Contrast		
	0	2	4	6	SE	*p*-Value	L	Q	C
pH	6.583	6.533	6.516	6.766	0.063	0.053	0.082	0.033	0.427
Molar proportion (mol/100 mol)									
Acetic	42.2	42.6	41.1	40.7	0.76	0.397	0.172	0.627	0.470
Propionic	26.3 ^bc^	27.9 ^ab^	30.4 ^a^	24.6 ^c^	0.38	0.006	0.235	0.002	0.012
Isobutyric	3.75 ^b^	3.56 ^b^	3.85 ^b^	4.56 ^a^	0.08	0.012	0.006	0.014	0.878
Butyric	18.7 ^a^	17.9 ^ab^	16.2 ^b^	19.2 ^a^	0.32	0.022	0.865	0.009	0.032
Isovaleric	5.81 ^b^	5.49 ^b^	5.68 ^b^	7.91 ^a^	0.11	0.001	0.001	0.001	0.059
Valeric	3.04	2.44	2.52	2.93	0.29	0.504	0.868	0.189	0.820

^a, b, c^ Columns with different letters indicate statistical difference (*p* < 0.05); SE: standard error; L: linear; Q: quadratic; C: quadratic.

**Table 5:** Protozoa population (log10 cells/mL) of growing lambs fed with different levels of olive oil.

Items	Olive Oil % DM					Significance			
							Contrast		
	0	2	4	6	SE	*p*-Value	L	Q	C
Holotrics (log 10)	2.942	2.758	3.133	3.222	0.140	0.144	0.076	0.349	0.202
Entodinium (log10)	4.436	4.601	4.086	4.130	0.215	0.313	0.161	0.784	0.222
Total protozoa (log10)	7.379	7.359	7.219	7.352	0.224	0.955	0.829	0.740	0.703

L: linear; Q: quadratic; C: cubic; SE: standard error.

**Table 6:** Production performance in lambs fed with different levels of olive oil.

Items	Olive Oil % DM					Significance			
							Contrast		
	0	2	4	6	SE	*p*-Value	L	Q	C
IW (kg)	13.0	13.1	13.3	12.2	1.43	0.958	0.759	0.692	0.839
FW (kg)	24.6	25.6	25.7	22.8	2.15	0.770	0.595	0.388	0.825
TWC (kg)	11.6	12.5	12.4	10.6	0.89	0.435	0.434	0.158	0.836
DWG (kg/day)	0.17	0.18	0.18	0.16	0.01	0.597	0.771	0.184	0.821
FC (kg/kg)	6.93	6.57	6.58	8.47	0.84	0.366	0.242	0.203	0.697

SE: standard error; L: linear; Q: quadratic; C: cubic. Total weight change; TPC: DWG; Daily Weight Gain; IW: initial weight; FW: final weight; DWG: daily weight gain; FC: feed conversion.

## Data Availability

Data will be available upon request to the corresponding authors.
